# Nuclei isolation from rat and cow white adipose tissues for single-nucleus RNA sequencing; rat WAT remains a challenge

**DOI:** 10.3389/fphys.2026.1741037

**Published:** 2026-03-26

**Authors:** Janice M. Thompson, Miguel Chirivi, Leah Terrian, G. Andres Contreras, Stephanie W. Watts, Rance Nault

**Affiliations:** 1 Department of Pharmacology and Toxicology, Michigan State University, East Lansing, MI, United States; 2 Department of Large Animal Clinical Sciences, Michigan State University, East Lansing, MI, United States; 3 Department of Biomedical Engineering, Michigan State University, East Lansing, MI, United States; 4 Institute for Integrative Toxicology, Michigan State University, East Lansing, MI, United States

**Keywords:** adipose tissue, cattle, nuclei isolation, rats, single-cell gene expression analysis

## Abstract

Perivascular adipose tissue (PVAT) is a complex tissue that is increasingly recognized for its roles in vascular health and disease. The form and function of PVAT are different depending on species and anatomical location, and understanding its cellular and molecular characteristics gives greater insights. We had previously successfully performed single-nucleus RNA sequencing (snRNAseq) on brown fat depots, the thoracic aortic PVAT (taPVAT) and subscapular brown adipose tissue (BAT), from Dahl Salt Sensitive rats. However, the application of the same nuclei isolation method to white adipose tissue (WAT) depots (perivascular and non-perivascular) from the same rat strain resulted in insufficient nuclei capture and low transcript numbers. These challenges were also encountered when processing WAT from cattle. While nuclei isolation methods have been developed and optimized for human and mouse WAT depots, they have not been evaluated across WAT depots from other species, such as cow and rat. Because these latter species are important models for cardiovascular and metabolic diseases, this study aimed to validate and optimize a nuclei isolation protocol for use with WAT from them. Protocols were evaluated based on a) the quantity of nuclei isolated, b) the quality of nuclei determined via microscopic visualization, and c) the total number of detected transcripts and genes following snRNAseq. A modified protocol developed for human WAT, incorporating liquid nitrogen pulverization and Dounce homogenization of flash-frozen tissue, was tested. This protocol, with key modifications for optimization, proved translatable to rat and cow WAT depots to improve nuclei yield (rat retroperitoneal fat, 3,100 nuclei/mg tissue; rat mesenteric perivascular adipose tissue, 2,200 nuclei/mg tissue; cow white fat, 2,050 nuclei/mg tissue). Further analysis via snRNAseq, however, identified limitations. While cow WAT expressed nearly 1,720 median genes/cell, both rat white depots were significantly lower [mesenteric PVAT (mesPVAT), 189; retroperitoneal (RP) fat, 294 median genes/cell], hindering downstream analyses in the rat tissue. These findings suggest that biological differences in adipose depots within and between species pose important challenges for the application of snRNAseq on rat WAT.

## 1 Introduction

Single-nucleus RNA sequencing (snRNAseq) is used to study the transcriptome of individual cells, relying solely on RNA from the isolated nuclei. This has been performed in a wide variety of tissues and across multiple species (Han et al., 2025; Kim et al., 2023). Nuclei can be obtained from fresh or frozen tissues, allowing for the use of archived samples. In addition, some tissues are dense, contain interconnected structures, or have a higher fat content, making them more difficult to process. SnRNAseq has proven reliable for these tissues, including the heart (Arduini et al., 2025), the brain (Lake et al., 2018), and tumors (Slyper et al., 2020). When working with different tissues and species, nuclei isolation techniques regularly require optimization (Kersey et al., 2025; So et al., 2025).

Our laboratories have previously published a cell atlas of thoracic aortic perivascular adipose tissue (taPVAT) from the Dahl SS rat, with a comparison to the non-vascular subscapular brown adipose tissue (BAT) (Thompson et al., 2024). When this protocol was used to evaluate rat white adipose tissue depots, both the mesenteric perivascular adipose tissue (mesPVAT) and non-vascular retroperitoneal (RP) fat, the number of captured nuclei was much lower than expected, with a small number of median detected genes per nucleus in mesPVAT (201 median genes; **Supplementary Figure 1A**).

White adipocytes contain larger lipid droplets in comparison to brown adipocytes (Kotzbeck et al., 2018; Rosell et al., 2014). Because of this, a different approach is needed to isolate sufficient nuclei and obtain sufficient RNA yield for analysis via snRNAseq. While human and mouse white adipose tissue have been evaluated in many studies (Emont et al., 2022; Van Hauwaert et al., 2021), and protocols benchmarked for different tissues and adipose depots within a species (So et al., 2025), the evaluation of protocol translation across species such as rat and cow, important cardiovascular and metabolic disease models, has not been described to our knowledge. These collective reasons prompted the development and validation of a modified protocol shared here.

In this study, the evaluation of a nuclei isolation protocol for white adipose depots from rats and cows, adapted from the protocol by Whytock et al. (2023), was described. For rat white adipose tissue (WAT), the perivascular adipose tissue (PVAT) surrounding the vessels of the mesentery was compared to retroperitoneal fat, also a WAT. PVAT from the rat thoracic aorta was used as a control for non-lipid-rich samples. For cows, the subcutaneous WAT from the paralumbar region was used. We show that the protocol was able to increase the nuclei yield for subsequent snRNAseq applications but did not lead to the detection of sufficient genes for downstream analysis and discovery for certain adipose depots, such as the mesPVAT.

## 2 Methods

### 2.1 Tissue collection

Male Dahl SS rats (RRID: RGD_1582190), 250–300 g, were purchased from Charles River Laboratories. Animals were fed a normal diet (Teklad) with food and drinking water available *ad libitum*. Rats were housed with a 12:12-h light:dark cycle. Animal procedures complied with the National Institutes of Health Guide for the Care and Use of Laboratory Animals (2011) and were approved by the Michigan State University Institutional Animal Care and Use Committee (PROTO202000009). Rats were given pentobarbital (Fatal-Plus; 80 mg/kg, i.p.) as a deep anesthetic, and a bilateral pneumothorax was created prior to tissue collection. Tissues were dissected and placed in beakers containing physiological salt solution [PSS; in mM: NaCl 130, KCl 4.7, KH_2_PO_4_ 1.18, MgSO_4_*7H_2_O 1.17, NaHCO_3_ 14.8, dextrose 5.5, CaNa_2_EDTA 0.03, and CaCl_2_ 1.6 (pH 7.2)]. The following tissues were dissected: thoracic aorta, subscapular (brown) fat, mesentery, and retroperitoneal fat. Following dissection, tissues were placed in a Silastic-coated dish containing PSS and cleaned of blood. For subscapular (brown) fat, care was taken to remove as much adjacent white fat as possible. Thoracic aorta PVAT was removed from the aorta, and mesenteric PVAT was removed from the resistance artery/vein arcades. Tissues were snap-frozen in liquid nitrogen in 1.5-mL microcentrifuge tubes and stored at −80°C until nuclei isolation was performed. **Tables 1A**-**E** list the key resources.

**Table 1A:** Animals.

Item	Source	Used in “Methods” section
Dahl SS male rats	Charles River Laboratories	2.1, S1
Teklad 22/5 rodent diet	Teklad	2.1, S1
Fatal-Plus	Vortech Pharmaceuticals	2.1, S1
Cows	Michigan State University Dairy Cattle Teaching and Research Center	2.1, S1

**Table 1B:** Reagents.

Reagent	Source	Catalog #	Used in “Methods” section
Liquid nitrogen	Airgas		2.1, 2.2
Sodium chloride (NaCl)	VWR	BDH9286	2.1
Potassium chloride (KCl)	Avantor	3040-05	2.1
Potassium phosphate, monobasic (KH_2_PO_4_)	Sigma-Aldrich	795488	2.1
Magnesium sulfate heptahydrate (MgSO_4_*7H_2_O)	VWR	0662-500G	2.1
Sodium bicarbonate (NaHCO_3_)	Sigma-Aldrich	S6014	2.1
Dextrose	Sigma-Aldrich	D9434	2.1
Ethylenediaminetetraacetic acid (EDTA), disodium salt, dihydrate	J.T. Baker	8993-01	2.1
Calcium chloride (CaCl_2_)	Sigma-Aldrich	C7902	2.1
Dithiothreitol	GoldBio	DTT10	2.2
Sucrose	Sigma	S-7903	2.2
10% Bovine serum albumin (BSA)	Millipore-Sigma	A1595-50mL	2.2, S1
Molecular Grade Nuclease-Free Water	Invitrogen	AM9930	2.2
Triton X-100	Sigma	T8787	2.2
Magnesium chloride	Sigma	M8266	2.2
Trizma base	Sigma	T1503	2.2
Potassium chloride	Avantor	3040-05	2.2
Halt protease inhibitor 100X	Thermo Scientific	87785	2.2
SUPERase·In (20 U/µL)	Invitrogen	AM2696	2.2
RiboLock RNAse Inhibitor	Thermo Scientific	EO0382	2.2, S1
DNase I	Qiagen	79254	2.2
Ice			2.2
Phosphate-buffered saline (PBS) without calcium and magnesium	Millipore-Sigma	D8537-500ML	2.2, S1
ViaStain AOPI stain	Nexcelom	CS2-0106-5mL	2.3, S1
Parse Evercode Nuclei Fixation kit	Parse Biosciences	ECF2003	2.4
Parse Evercode WT Mini kit	Parse Biosciences	ECW02010	2.5
Qubit dsDNA HS (high sensitivity) Assay kit	Invitrogen	Q33230	2.5
Qiagen RNeasy Plus Universal Kit	Qiagen	73404	S1
TapeStation High Sensitivity RNA Screen Tape	Agilent		S1
Minute Nuclei and Cytosol Isolation Kit for Adipose Tissues/Cultured Adipocytes	Invent Biotechnologies	AN-029	S1
10X Genomics 3′ v3.1 Chromium chip	10X Genomics		S1
10X Genomics Next GEM Single Cell 3′ Reagent Kit v 3.1 (Dual Index)	10X Genomics		S1
KAPA Library Quantification Kit	KAPA Biosystems		S1

**Table 1C:** Consumables.

Item	Source	Catalog #	Used in “Methods” section
2-mL LoBind microcentrifuge tubes	Eppendorf	022431048	2.2
Weigh boats	VWR	10770-442	2.2, S1
Mortar and pestle	Varies (ex. Cole Parmer)		2.2
Spatula	Varies (ex. Fisher Scientific)		2.2
Glass Dounce homogenizer	Varies (ex. Corning)		2.2
1.7-mL protein LoBind microcentrifuge tubes	Eppendorf	022431021	2.2
20 µL low binding pipette tips	Rainin	30389226	2.2
200 µL low binding pipette tips	Rainin	30389240	2.2
1,000 µL low binding pipette tips	Rainin	30389213	2.2
100-µm filters	VWR	76327-102	2.2
40-µm filters	VWR	76327-098	2.2
20-µm filters	Pluriselect-usa, Inc.	43-50020	2.2
15-mL conical tubes	Corning	430052	2.2
50-mL conical tubes	Corning	430290	2.2
Gel loading tips	Fisherbrand	02-707-181	2.2
1-mL syringe with 25-gauge needle	Becton Dickinson	309626	2.2
PCR strip tubes	Genessee Scientific	27-125	2.4
SPRIselect beads	Beckman Coulter	19686100	2.4, 2.5
20 µm Pluri-Select cell strainer	Pluriselect-usa, Inc.	43-10020-40	2.5
50-mL reservoirs	Costar	4870	2.5
FLOWMI 40 µm cell strainer	SP Bel-Art	136800040	S1

**Table 1D:** Equipment.

Item	Source	Used in “Methods” section
Balance	Varies (ex. Mettler)	2.1
Vortex	Varies (ex. Scientific Industries)	2.2, 2.5
Plate adapter for vortex	Varies (ex. Scientific Industries)	2.5
Hemacytometer	Varies (ex. Thermo Fisher)	2.3
Cellometer Spectrum	Nexcelom Bioscience	2.3, S1
Microscope for brightfield and fluorescence	Varies (ex. Nikon)	2.3
Mr. Frosty (or comparable freezing container)	Varies (ex. Thermo Fisher)	2.4
Refrigerated centrifuge with swinging bucket rotor	Varies (ex. Beckman Coulter)	2.4, 2.5
SimpliAmp ThermalCycler	Applied Biosystems	2.5
Qubit	Thermo Fisher	2.5
Agilent 4200 TapeStation	Agilent Technologies	2.5
D5000 Screen Tape Assay	Agilent Technologies	2.5
D1000 Screen Tape Assay	Agilent Technologies	2.5
NovaSeq6000	Illumina	2.5
BD FACSAria IIu	Becton Dickinson	S1

**Table 1E:** Analysis tools.

Program	Source	RRID	Used in “Methods” section
Spectrum Cell Analyzer Software V 3.2.0.11	Nexcelom Bioscience		2.3
split-pipe v1.5.1	https://www.parsebiosciences.com		2.6
FastQC v0.11.7	github.com/s-andrews/FastQC	SCR_014583	2.6
scanpy v1.9.1	github.com/scverse/scanpy	SCR_018139	2.6
scDblFinder v1.14.0	github.com/plger/scDblFinder	SCR_022700	2.6
scvi-tools v0.20.0	github.com/scverse/scvi-tools	SCR_026673	2.6
10X Genomics CellRanger v7.1.0	https://www.10xgenomics.com/support/software/cell-ranger/latest		S1

Lactating Holstein dairy cows from the Michigan State University Dairy Cattle Teaching and Research Center were housed in individual tie-stalls bedded with sawdust (cleaned twice daily), were milked three times daily (at 04:00, 12:00, and 20:00 h), and had water available *ad libitum*. All animals received a common lactation diet formulated to meet their energy requirements (National Academies of Sciences, Engineering, and Medicine et al., 2021). All experimental procedures were approved by the Institutional Animal Care and Use Committee (PROTO202100042) at Michigan State University (East Lansing, MI, USA). Subcutaneous WAT samples (~1 g) were collected from the right paralumbar fossa cranial to the tensor fasciae latae muscle, the right flank, following our established protocol (Chirivi et al., 2025). The cows were 15 days in milk in their second lactation and were confirmed to be clinically healthy, showing no evidence of metabolic or infectious disorders at the time of sampling.

### 2.2 Nuclei isolation

#### 2.2.1 Notes before beginning

Obtain reagents, consumables, and equipment as indicated in **Tables 1B**, **1C**, and **1D** for Section 2.2 (“Nuclei Isolation”). Prepare buffer reagents as indicated in the “Reagents” section and store according to recommendations. Freshly prepare homogenization and nuclei resuspension buffers (**Tables 2**, **3**) daily. Do not store for reuse. Keep on ice and protected from light. Obtain liquid nitrogen and frozen tissue samples.

**Table 2:** Homogenization buffer.

Reagent	Volume (µL)	Sample #	Total volume (µL)	Final concentration
1 M MgCl_2_	15			5 mM
1 M Tris Buffer, pH 8.0	30			10 mM
2 M KCl	37.5			25 mM
1.5 M sucrose	500			250 mM
1 mM DTT	3			1 µM
Halt Protease Inhibitor (100X)	30			1×
SUPERase·In (20 U/µL)	30			0.2 U/µL
Nuclease-free water	2354.5			N/A

Total volume, 3,000 µL.

**Table 3:** Nuclei resuspension buffer.

Reagent	Volume (µL)	Sample #	Total volume (µL)	Final concentration
1 M MgCl_2_	10			5 mM
1 M Tris Buffer, pH 8.0	20			10 mM
2 M KCl	25			25 mM
0.5 M EDTA	4			1 mM
RiboLock RNAse Inhibitor	20			0.2 U/µL
DNase I	14.7			40 units
1% BSA nuclease-free water	1906.3			

Total volume, 2,000 µL.

#### 2.2.2 Reagents

- 1 mM dithiothreitol (DTT): Add 1 mg DTT to 2.82 mL nuclease-free water and dissolve. Aliquot 20 µL to 0.5-mL tubes and store at −20°C.
- 1.5 M sucrose: Add 20.54 g sucrose to 40 mL nuclease-free water and dissolve. Aliquot 1 mL to 1.5-mL tubes and store at −20°C.
- 1% bovine serum albumin (BSA) nuclease-free water: Add 5 mL 10% BSA to 45 mL nuclease-free water. Filter through a 0.2-µm syringe filter and store at 4°C.
- 10% Triton X-100: Add 100 µL 100% Triton X-100 to 900 µL nuclease-free water and dissolve. Store at room temperature.
- 1 M magnesium chloride (MgCl_2_): Add 9.5211 g MgCl_2_ to 90 mL nuclease-free water and dissolve. Bring the final volume to 100 mL and store at room temperature.
- 1 M Tris Buffer, pH 8.0: Add 12.114 g Trizma base to 90 mL nuclease-free water and dissolve. Adjust pH to 8.0. Bring the final volume to 100 mL and store at room temperature.
- 2 M potassium chloride (KCl): Add 14.91 g KCl to 80 mL nuclease-free water and dissolve. Bring the final volume to 100 mL and store at room temperature.
- 0.5 M ethylenediaminetetraacetic acid (EDTA): Add 1.46 g EDTA to 8 mL nuclease-free water. Bring the final volume to 10 mL and store at room temperature.

Prepare homogenization buffer (**Table 2**)—Keep on ice and protect from light.

The table identifies the amount needed for one sample. Space is provided in the table for the calculation of specific numbers of samples and volumes.

Prepare nuclei resuspension buffer (**Table 3**)—Keep on ice and protect from light.

The table identifies the amount needed for one sample. Space is provided in the table for the calculation of specific numbers of samples and volumes.

#### 2.2.3 Tissue pulverization and homogenization

**Figure 1:**
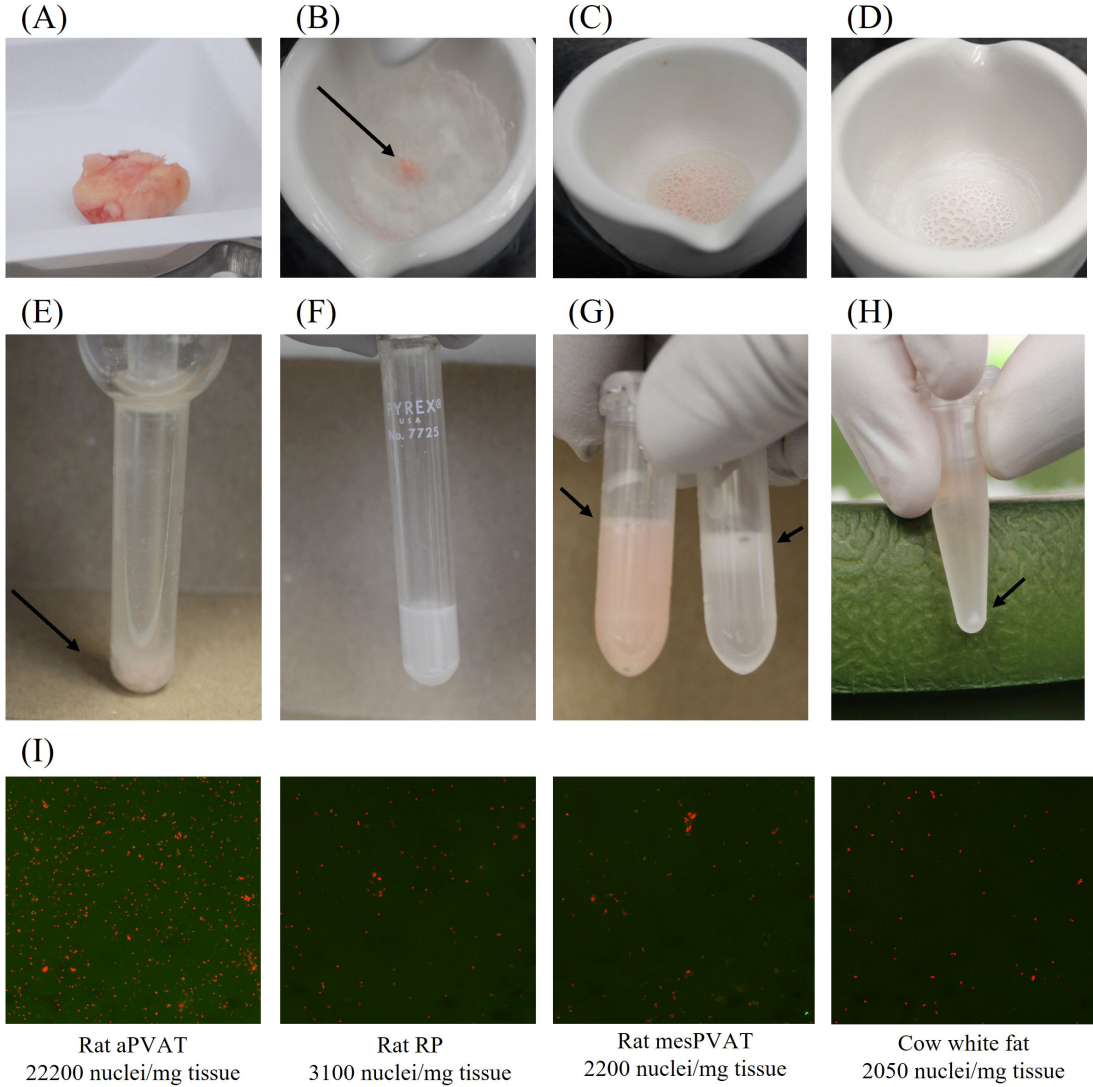
Representative images from nuclei isolation methods. **(A)** Tissue before pulverization, **(B)** tissue in liquid nitrogen, **(C)** pulverized tissue with liquid nitrogen, **(D)** pulverized tissue after liquid nitrogen evaporation, **(E)** sample before Dounce homogenization, **(F)** sample after Dounce homogenization, **(G)** samples in LoBind tubes before centrifugation, **(H)** samples after final centrifugation, and **(I)** AO/PI-stained nuclei from Cellometer. Images are representative of tissue types used for nuclei isolation. AO/PI, acridine orange/propidium iodide.

This protocol was adapted from a human subcutaneous adipose tissue isolation published by Whytock et al. (2023). One sample at a time was pulverized and homogenized, with a maximum of four samples processed at one time to avoid sample degradation. *Mortar, pestle, and spatula were cooled with liquid nitrogen. *Caution: When handling liquid nitrogen, ensure that appropriate personal protective equipment is used.* Liquid nitrogen was poured into a mortar, and the pestle was placed inside to ensure adequate cooling for both pieces. When liquid nitrogen was fully evaporated from the mortar, another volume was added. While cooling, an appropriate amount of tissue was weighed (**Figure 1A**), and the values were recorded. For rats, approximately 150 mg of tissue was used. For cows, approximately 350 mg of tissue was used. The weighed tissue was added to the mortar when the second volume of liquid nitrogen was added (**Figure 1B**). The tissue was tapped gently with the pestle as the liquid nitrogen evaporated to break into smaller pieces (**Figure 1C**). Following liquid nitrogen evaporation, the tissue was pulverized thoroughly, with care taken to keep all tissue pieces in the mortar (**Figure 1D**). Using a spatula, the frozen mixture was transferred into a glass Dounce homogenizer, 2 mL of homogenization buffer was added (**Figure 1E**), and the sample was homogenized with 10 strokes (**Figure 1F**). The homogenate was transferred equally to two 1.7-mL protein LoBind tubes and placed on ice. Four hundred microliters of homogenization buffer was added to the Dounce homogenizer to retrieve residual homogenate, and the buffer was added to the two sample tubes in equal volumes.* The process indicated in the “Tissue Pulverization and Homogenization” section, from * to *, was repeated until all samples were processed, with homogenates on ice during this time.

#### 2.2.4 Nuclei isolation

Triton X-100 (10%, 12 µL) was added to each 1.7-mL sample tube. Tubes were pulse-vortexed for 2 seconds, incubated on ice for 30 minutes, and covered to protect from light, with pulse vortexing every 5 minutes. While samples were incubating, filters were prepared: for the cow samples, 100-, 40-, and 20-µm filters were placed in separate 50-mL conical tubes, and each filter was pre-wetted with 100 µL of phosphate-buffered saline (PBS). For each rat sample, 100- and 40-µm filters were placed in separate 50-mL conical tubes, and each filter was pre-wetted with 100 µL of PBS. The 20-µm filter was omitted from rat samples to prevent excess sample loss. When incubation was complete, samples were filtered: the homogenate was pipetted through a 100-µm filter into the 50-mL conical tube. Homogenization buffer (400 µL) was added to the 1.7-mL tubes as a wash. The wash was collected and pipetted through the 100-µm filter. Homogenate from the 50-mL conical tube was pipetted through the 40-µm filter and then through the 20-µm filter (for cows) into its 50-mL conical tubes, and the homogenate was aliquoted equally to two 1.7-mL LoBind tubes (~1.1 mL per tube). Rat taPVAT homogenates were centrifuged at 500 × *g* for 10 minutes, while bovine and rat WAT samples were centrifuged at 2,700 × *g* for 10 minutes at 4°C. Following centrifugation, the lipid layer (**Figure 1G**) was removed using a gel loading tip. The remaining supernatant was pipetted off and discarded, leaving ~50 µL, and the tubes were placed on ice. Homogenates were combined into one tube for each sample: the pellet in the first tube was resuspended in its 50-µL volume by pipetting up and down 30 times and then transferred to a new 1.7-mL protein LoBind tube (Tube 1). The pellet in the second tube was resuspended in its 50-µL volume by pipetting up and down 30 times and transferred to Tube 1, resulting in pooled homogenates for each sample. One hundred microliters of homogenization buffer was added to the pooled homogenate in Tube 1, mixed by pipetting five times, and then centrifuged at 500 × *g* (rat taPVAT) or 2,700 × *g* (bovine WAT and rat WAT) for 10 minutes at 4°C. Note: There should be visible pellets and no lipid at the top. Supernatants were removed and discarded, leaving ~50 µL on top of each pellet. Pellets were resuspended by pipetting up and down 30 times in the ~50-µL volume.

To each sample, 950 µL nuclei resuspension buffer was added and mixed by pipetting up and down 10 times. Samples were centrifuged at 500 × *g* (rat taPVAT) or 2,700 × *g* (bovine WAT and rat WAT) for 10 minutes at 4°C (**Figure 1H**). Centrifugation speed and duration were determined using test samples by examining the number of nuclei present in the pellet compared to the supernatant, clumps remaining, and nuclei integrity. Supernatants were removed, leaving ~50 µL on top of each pellet. Pellets were resuspended in the 50-µL volume by pipetting up and down 30 times. Nuclei resuspension buffer (450 µL) was added to each sample and mixed by pipetting up and down 10 times. To reduce clumping, the nuclei solution was pulled up and down 10 times using a 25G needle with a 1-mL syringe, carefully avoiding the introduction of bubbles. Samples were placed on ice while staining for nuclei count/integrity.

### 2.3 Nuclei counting

Notes: When counting nuclei, move quickly and ensure that samples remain on ice to prevent degradation. In general, ≤30 minutes on ice can reduce clumping or lysing of nuclei. If using a hemacytometer and microscope, take representative pictures at low and high magnification of nuclei, as well as on brightfield, for archiving. If using an automated cell counter, such as a Cellometer Spectrum (Nexcelom Bioscience, Spectrum Cell Analyzer Software V 3.2.0.11), ensure that images are taken on both the fluorescent and brightfield channels. These images (from the microscope of an automated cell counter) can also be helpful to assess nuclei quality or estimate clumping or low counts following the fixation step, as well as assess debris contamination. While the hemacytometer/microscope method is recommended for nuclei counting for higher accuracy, automated cell counting is acceptable. For this step, we used an automated cell counter due to the lack of an available microscope.

For each nuclei isolation sample, acridine orange/propidium iodide (AO/PI) stain was added 1:1 in a new 0.5-mL microcentrifuge tube and then pipetted up and down 10 times to mix. (Optional: 4′,6-diamidino-2-phenylindole (DAPI) can be used in place of AO/PI; however, it did not stain white fat nuclei as well as AO/PI during our experiments.) Each sample was counted using a Nexcelom Cellometer (**Figure 1I**). Following counting, samples were immediately processed as indicated in the “Nuclei Fixation” section.

A step-by-step version of this protocol is also available on protocols.io (DOI: dx.doi.org/10.17504/protocols.io.kxygx42kwl8j/v1).

### 2.4 Nuclei fixation

Notes: The Parse Evercode Nuclei Fixation kit (Parse Biosciences, Seattle, WA, USA) was used for fixation of isolated nuclei, a process that fixes and permeabilizes nuclei for use, with the Evercode WT Mini kit Protein LoBind tubes used in place of blocking tubes with BSA.

Approximately 100,000 freshly isolated and counted nuclei samples were immediately processed using the Parse Evercode Nuclei Fixation kit following the manufacturer’s instructions. After fixation, all samples were counted using a Nexcelom Cellometer with the process identified in the “Nuclei Counting” section, and values were recorded. A 10 µL aliquot of each sample was placed in a separate 0.5-mL microcentrifuge tube for counting post-freezing, and all samples were placed in a Mr. Frosty freezing container at −80°C following the manufacturer’s recommendation until processed as indicated in the “Barcoding and Sequencing” section.

### 2.5 Barcoding and sequencing

Notes: The Parse Evercode WT Mini kit was used for barcoding and sequencing library preparation.

The counting samples indicated in the “Nuclei Fixation” section were thawed and counted using a hemacytometer and microscope (Grigoryev, 2025), and values were recorded; then, the appropriate volume of each nuclei suspension to be added to the barcoding plate was determined using the Parse Biosciences Evercode WT Mini Sample Loading Table V2. Barcoding was completed using the Parse Evercode WT Mini kit following the manufacturer’s directions as outlined in the Parse Evercode WT Mini v3 User Manual v1.5. The resulting libraries were submitted to Novogene for 150-bp paired-end sequencing at a target depth of 50,000 reads/nuclei.

### 2.6 Computational analysis

The quality of the raw sequencing files was first checked using FastQC. Reads were then split by species using the fastq_sep_groups_v0.5.py script from Parse (v0.5, Parse Biosciences, Seattle, WA, USA). Rat and cattle reads were aligned to their respective reference genomes (GRCr8, ARS-UCD1.3) using the Parse Biosciences split-pipe pipeline (v1.5.1, Parse Biosciences, Seattle, WA, USA). Alignment quality was assessed, and the resulting filtered features, genes, and matrix files (DGE_filtered) were further processed and analyzed using Python. First, the aligned reads were preprocessed using scDblFinder (Germain et al., 2021) to identify doublets. Then, individual samples were further preprocessed using the scanpy Python package (Wolf et al., 2018). Quality control cut-offs were similar to those recommended in the “Single Cell Best Practices” manual (Heumos et al., 2023). Genes found in three or fewer cells were removed, as were cells with fewer than 100 measured transcripts. Nuclei that had greater than 15,000 reads or 4,500 measured genes were also discarded. A mitochondrial read percentage cut-off of 3% was used since single-nucleus RNAseq is expected to capture far fewer mitochondrial reads than single-cell RNAseq. After individual preprocessing, the anndata objects for each sample were combined, and additional preprocessing was performed. Nuclei that were outside of five median absolute deviations from the medians of the number of detected genes (log1p_n_genes_by_counts) and number of detected RNA molecules (log1p_total_counts) were labeled outliers and discarded. The high-dimensional data were then visualized using principal component analysis (PCA) and Uniform Manifold Approximation and Projection (UMAP). Batch integration was performed using scvi-tools (Gayoso et al., 2022) to remove technical noise from the reduced-dimension plots. Clustering was performed using the Leiden algorithm. Previously published marker genes and cluster-specific genes identified via differential expression analysis were used to manually annotate each cluster.

## 3 Results

### 3.1 Homogenization and centrifugation speed modifications result in increased nuclei yield from rat and bovine white adipose tissue

In previous attempts to isolate nuclei from cow and rat white adipose tissues following a previously published protocol (**Supplementary File S1**; Truong et al., 2020), fewer than 200 nuclei/mg of tissue were recovered. This yield was insufficient for subsequent snRNAseq applications, particularly for depots with limited tissue mass (e.g., mesPVAT), necessitating the modifications identified here.

Rat and cow adipose tissue samples (representative image shown in **Figure 1A**) were pulverized in liquid nitrogen, followed by homogenization in a Dounce homogenizer containing a Tris/sucrose-based buffer. Removal of the lipid layer at the top of all fat homogenates was completed using gel loading tips, as their finer points enabled a more thorough evacuation. Centrifugation speeds for the rat taPVAT were 500 × *g*, while cow WAT and rat WATs required 2,700 × *g*. The pellet in **Figure 1H** is representative of all samples.

**Figure 1I** depicts isolated nuclei from rat taPVAT as adipose tissue reference producing sufficient nuclei concentrations, followed by rat RP fat, rat mesPVAT, and cow WAT. Nuclei were stained with AO/PI and imaged on a Nexcelom Cellometer. Counts were obtained and normalized to the amount of input tissue. All white fats showed an increase in nuclei isolated versus previous attempts, ranging from 2,050 nuclei/mg tissue in cow white fat to 3,100 nuclei/mg tissue in rat RP fat (**Figure 1I**).

**Figure 2:**
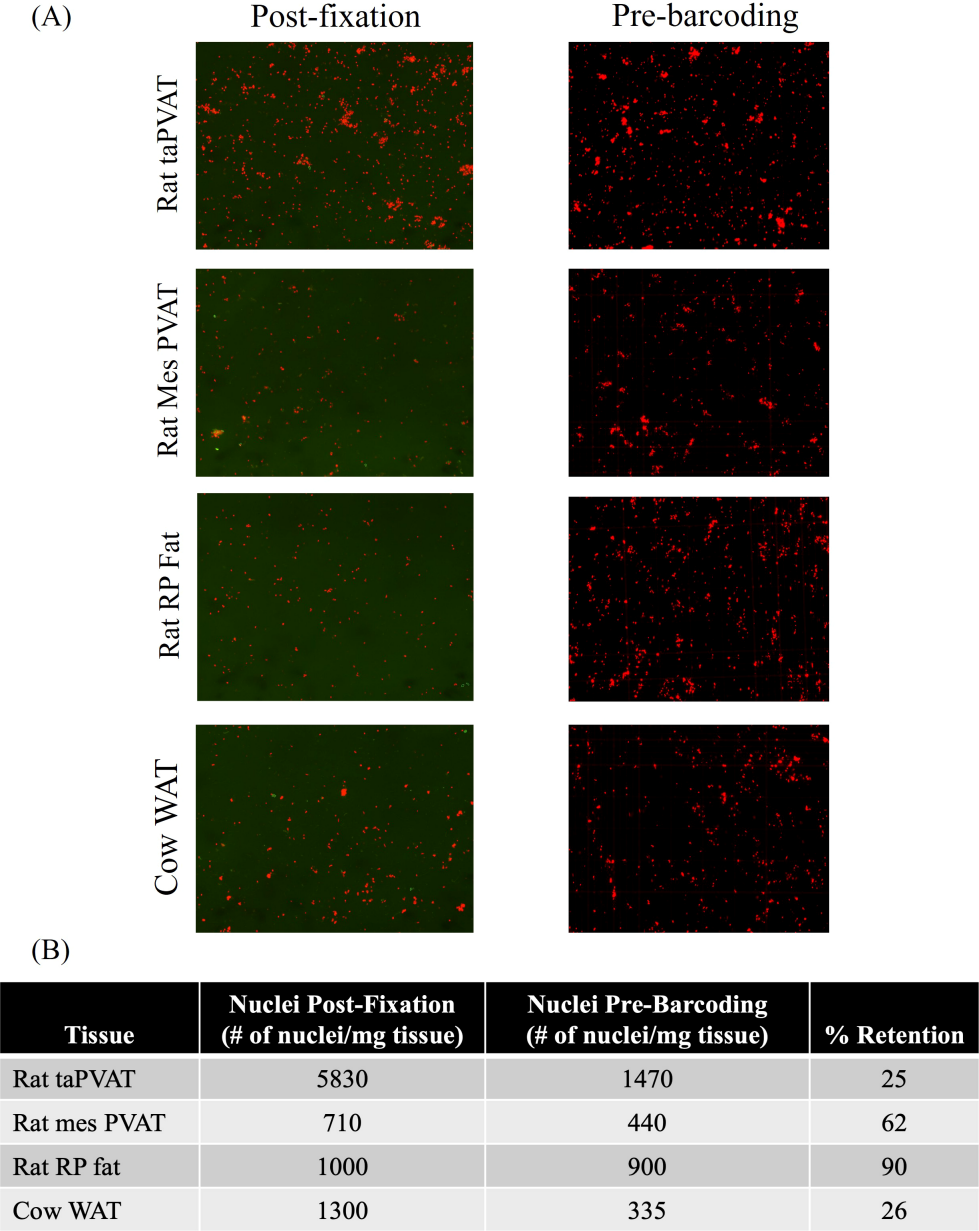
**(A)** Images of nuclei post-fixation (left column) and pre-barcoding (right column). Post-fixation images were captured using a Cellometer; pre-barcoding images were captured using a microscope. **(B)** Table identifies the number of nuclei per milligram of tissue at each step, followed by % retention.

With the number of nuclei in the required range for snRNAseq, the Parse Evercode Fixation kit was used to preserve them for snRNAseq analysis. Fixed nuclei were imaged (**Figure 2A**, left column), frozen at –80°C for 2 weeks, and then thawed and re-imaged (**Figure 2A**, right column). Retention of samples ranged from 26% in cow WAT to 90% in rat RP fat (**Figure 2B**).

**Figure 3:**
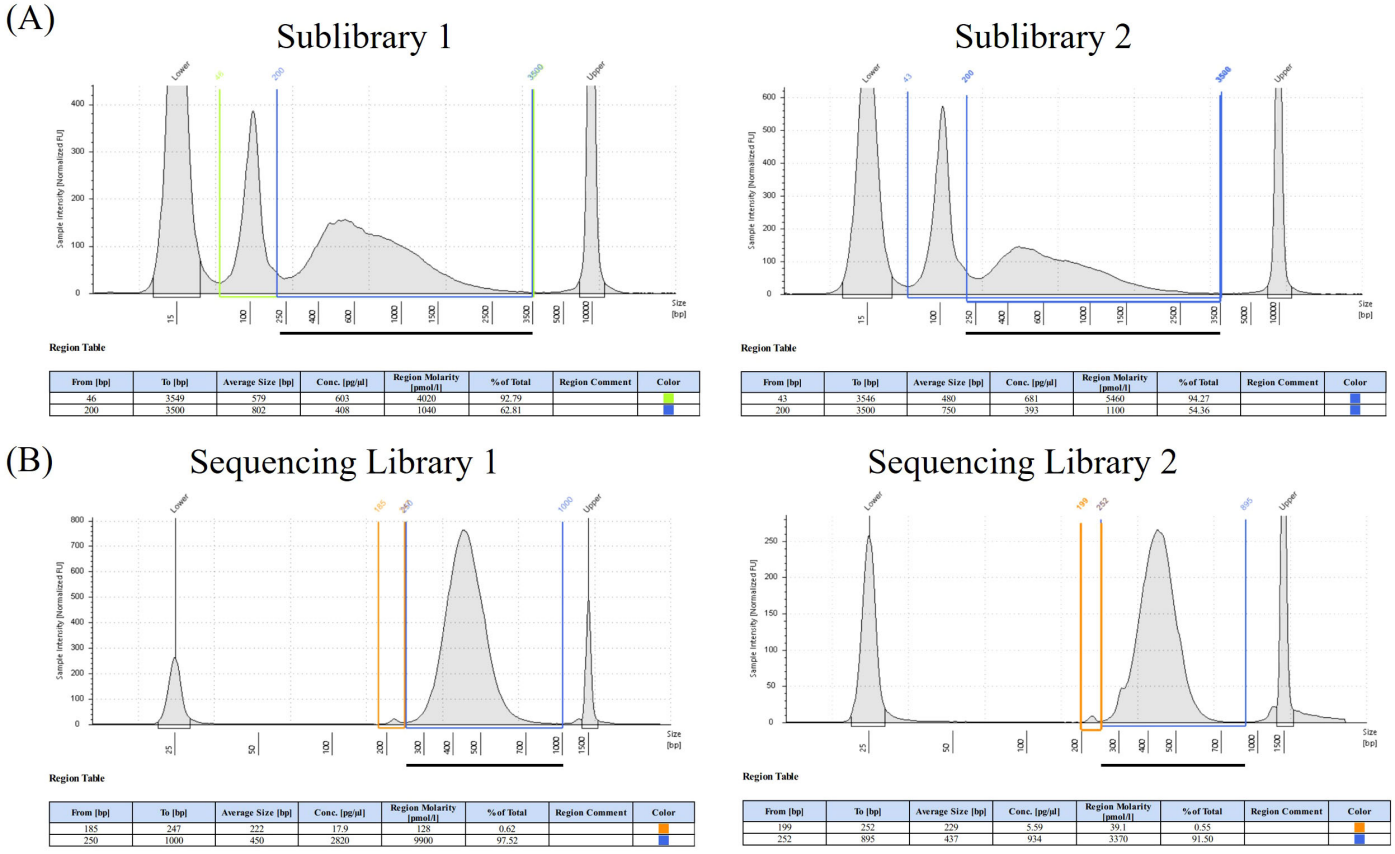
**(A)** Representative trace of sublibrary cDNA size distribution following first and second barcoding steps with the Parse workflow from sublibrary 1 and sublibrary 2, indicating reproducibility between samples. Analysis performed on an Agilent TapeStation system. Black line indicates region of interest. **(B)** Representative tracing of workflow from sequencing library 1 and sequencing library 2, indicating reproducibility between samples. Analysis performed on an Agilent TapeStation system. Black line indicates region of interest.

Two sublibraries were created from brown and white fat samples using the Parse Evercode WT Mini kit. Analysis using TapeStation D5000 (**Figure 3A**) identified similar peaks between 200 and 3,000 bp, indicating size distribution reproducibility between the final library samples. Two additional peaks were observed. The first, in the 25-bp range, was an artifact, as detection of the D5000 was from 100 to 5,000 bp. The second peak, at approximately 100 bp, indicated potential adapter dimerization, which was addressed in future steps. Following additional cleanup steps and a final round of barcoding, two sequencing libraries were created and analyzed using TapeStation D1000 (**Figure 3B**). Indeed, the adapter dimers were nearly eliminated in each library.

### 3.2 Low RNA content remains a challenge for rat WATs

**Figure 4:**
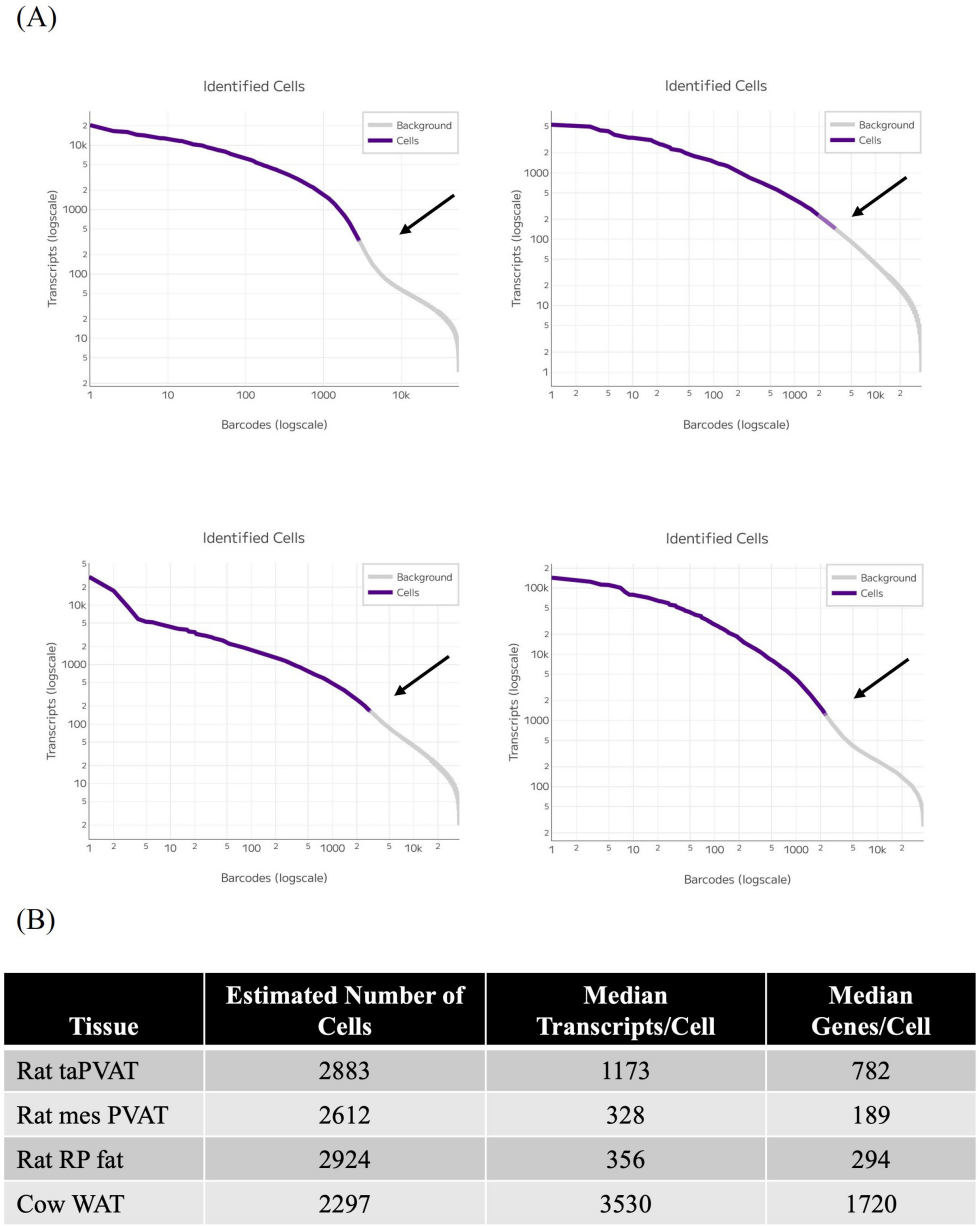
**(A)** Knee plot of taPVAT, as control, identifying distinct inflection point between real cells and background noise, such as ambient RNA, compared to RP fat, mesPVAT, and cow fat. Arrows indicate inflection point in taPVAT and no clear inflection point in RP fat or mesPVAT, but a moderate one in cow WAT. **(B)** Estimated number of cells, median transcripts/cell, and median genes/cell for all samples in panel **(A)** PVAT, perivascular adipose tissue; taPVAT, thoracic aortic PVAT; RP, retroperitoneal mesPVAT, mesenteric PVAT; WAT, white adipose tissue.

The quality control of sequencing reads was performed using FastQC, followed by the Parse Biosciences split-pipe pipeline (v1.5.1, Parse Biosciences, Seattle, WA, USA). The generated knee plots show a distinct inflection point in both rat taPVAT and bovine WAT. No clear inflection points were observed for the rat mesPVAT or RP fat, indicative of difficulties in resolving true nuclei from background/ambient RNA (**Figure 4**).

The estimated number of captured cells for each sample is reported (**Figure 4**), ranging from 2,297 in bovine WAT to 2,924 in RP fat for a targeted capture rate of 4,000 cells/sample. The median number of transcripts per cell, defined as the median count of all Unique Molecular Identifiers (UMIs) or reads, represents the amount of RNA detected. This includes multiple transcripts of the same gene. Cow WAT contained the most, at 3,530 median genes, while taPVAT contained 1,173 median genes. In contrast, rat mesPVAT contained only 328 median genes, while RP had 356 median genes, likely reflecting the difficulty in resolving true nuclei. The number of total UMI (transcripts) associated with a single nucleus was as high as ~5,000 in mesPVAT and ~30,000 in RP fat, showing that there are likely true nuclei represented in these WATs, as other depots, such as the taPVAT and BAT, were ~2,000 (Thompson et al., 2024). However, the small range of total UMI made it harder to separate them from the background. Additionally, the median genes per cell, a median count of unique genes per cell, is reported. Again, cow WAT contained the most, with 1,720, followed by taPVAT at 782. RP fat contained 294, while mesPVAT contained the least, with 189 (**Figure 4**).

**Figure 5:**
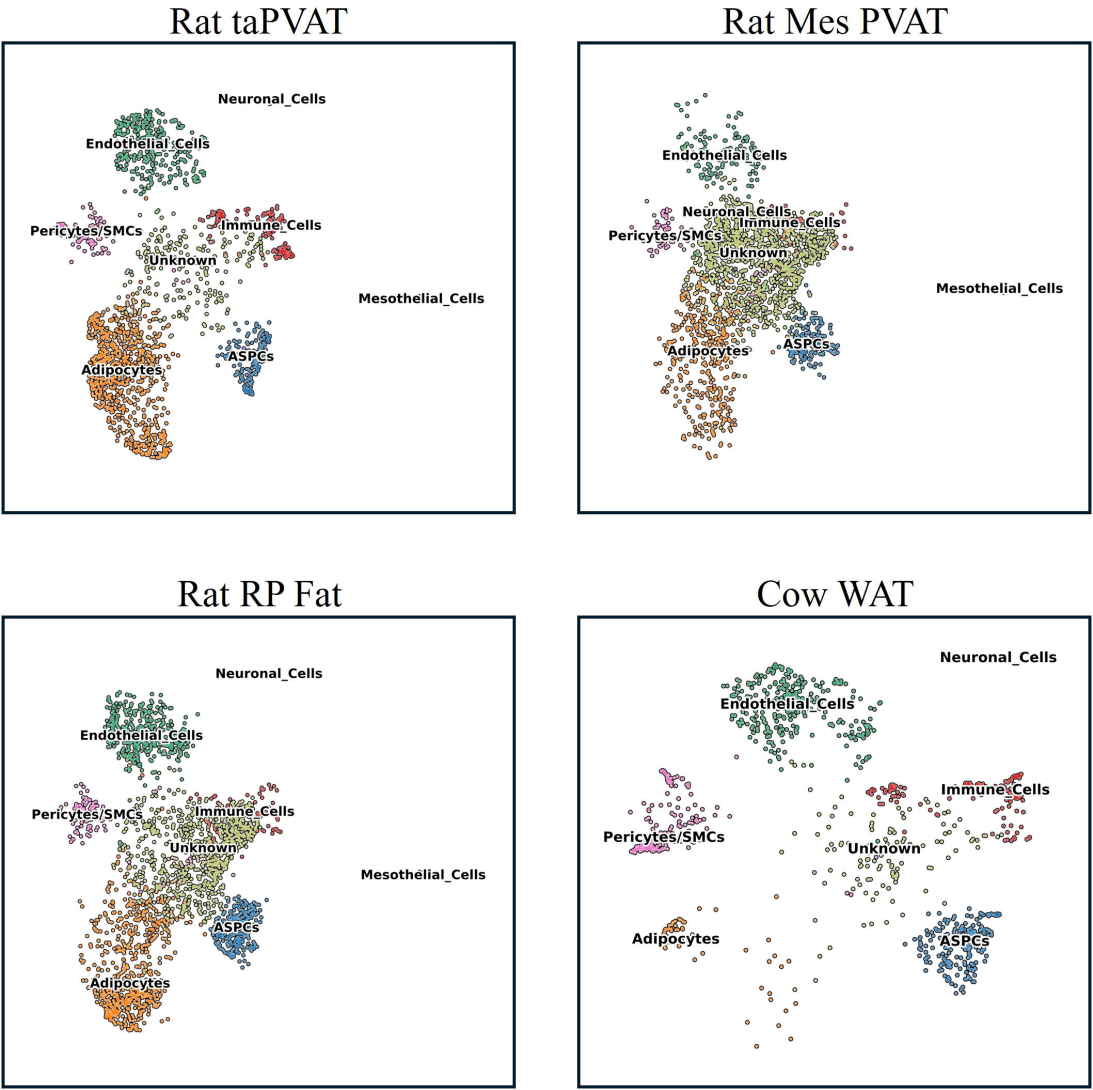
Uniform Manifold Approximation and Projection (UMAP) visualization of nuclei from rat taPVAT, RP fat, mesPVAT, and cow WAT following integration using single-cell variational inference (scVI) and manual annotation of cell types. PVAT, perivascular adipose tissue; taPVAT, thoracic aortic PVAT; RP, retroperitoneal; mesPVAT, mesenteric PVAT; WAT, white adipose tissue.

**Figure 6:**
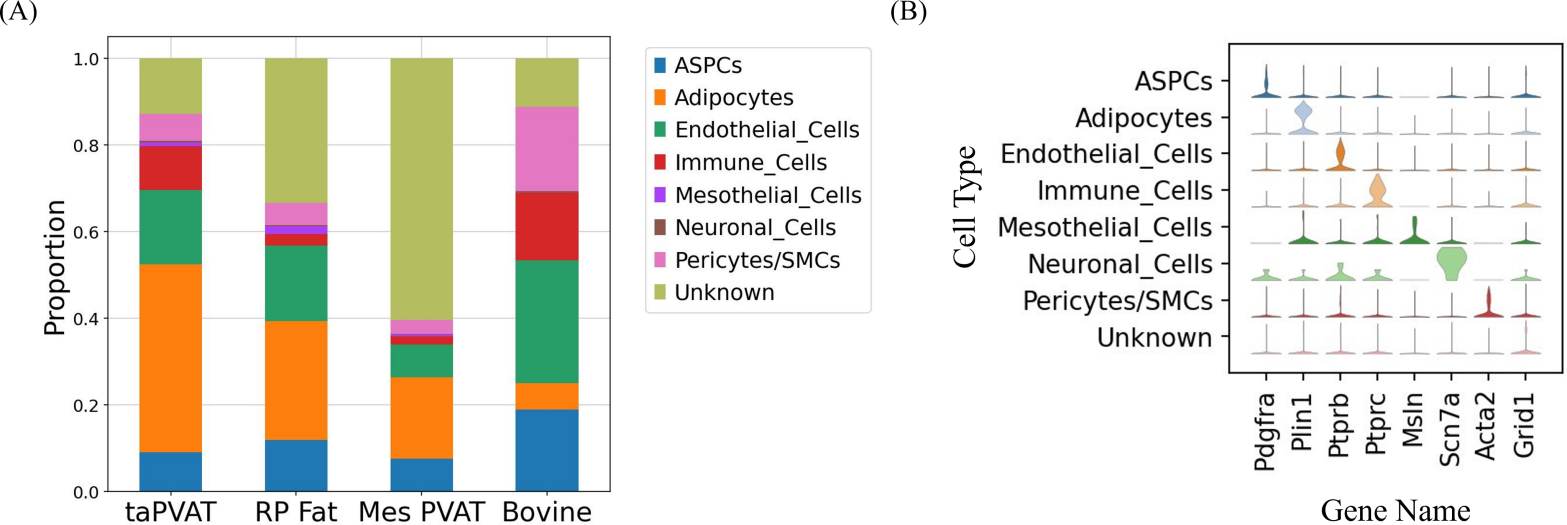
**(A)** Cell type composition by tissue. **(B)** Stacked violin plot of marker genes for each cell type annotation.

Manual annotation of the generated UMAPs and Leiden clusters reveals the presence of adipocytes in all samples, as well as endothelial cells, adipocyte stem and progenitor cells (ASPCs), and immune cells (**Figure 5**). A large number of nuclei were annotated as unknown in both rat mesPVAT and RP fat. **Figure 6A** shows this distribution, with the marker genes used to identify cell types shown in **Figure 6B**; **Supplementary 2**. Although the proportion of adipocytes in cow samples was small (~5%) compared to previous reports (Michelotti et al., 2022; Chi et al., 2024), adipocytes were the largest population found in all other adipose depots, suggesting that the sample may have had a smaller number rather than a technical artifact. The unknown cell clusters expressed clearly distinct markers, but none that could be used to confidently map to a known cell type. This is likely a consequence of the low median number of genes per nucleus in the rat WAT samples and not the identification of a novel cell population.

## 4 Discussion

Omics applications for adipose tissue are often complicated by the unique nature of fat depots, which consist of a significant population of lipid-laden adipocytes that are large, fragile, and buoyant. These characteristics make it particularly difficult to isolate cells for RNAseq applications, hence the use of snRNAseq when working with adipose tissue. The focus of this study was to optimize a method that improved the number of nuclei recovered from white adipose depots. The protocol shown here demonstrates that a sufficient number of nuclei can be isolated from all rat and cow white adipose tissues, a criterion needed to proceed with snRNAseq, in this case, using the Parse Evercode WT platform. This adaptation of a human subcutaneous fat protocol (Whytock et al., 2023) shows both the versatility and limitations of use for multiple adipose depots (taPVAT, retroperitoneal fat, mesPVAT, and paralumbar fossa white fat) and across species (rat and cow).

While the number of nuclei was sufficient for snRNAseq analysis, different sequencing results were obtained from the individual depots. Rat BAT and cow WAT detected a sufficient number of genes for downstream analyses. By contrast, rat WAT was below the threshold considered necessary for robust cell annotation and downstream analyses. Isolation of RNA from representative rat mesPVAT, collected and stored as the samples for snRNAseq, yielded RNA integrity numbers (RINs) from 7.0 to 7.9 (**Supplementary Figure 1A**), indicating that the RNA was not degraded prior to nuclei isolation. This suggests that RNA integrity is not likely to explain these differences. Moreover, these challenges were observed irrespective of downstream snRNAseq technology (**Supplementary Figure 1C**), indicating a more likely biological contributing factor rather than technical. Knowing this, we must determine if this is due to poor tissue quality, the nuclei isolation technique, or something inherent in the tissue itself, such as a minimal amount of RNA.

The substantial increase in the number of nuclei obtained in this isolation technique indicates that it is possible to obtain sufficient numbers from each rat WAT. However, this still did not yield better results when it came to the sequencing. We could not identify experimental interventions that resulted in the low RNA yield, and thus, we speculated that the low yield must be inherent to these two rat WATs. A large difference in the amount of RNA between BAT and WAT is reported (Kurabo Industries, Ltd., 2006), and adipose tissues have generally been considered challenging tissues for RNA-based studies due to the high amounts of lipids (Hemmrich et al., 2010; Cirera, 2013). Previous studies have suggested that the proximity of the mesPVAT to the pancreas can lead to tissue contamination and RNA degradation (Caesar and Drevon, 2008). However, our higher RIN estimates and the absence of pancreatic cell markers in the snRNAseq led us to believe that this is not the case.

Our analyses with rat RP fat and mesPVAT isolated nuclei have been evaluated on both the 10X Genomics and Parse Biosciences platforms, yielding consistent results. While knee plots from our evaluation using the 10X Genomics platform (**Supplementary Figure 1C**) show moderate inflection points for mesPVAT samples and a distinct one for the RP fat, the median number of genes for RP fat was 1,866, and mesPVAT was an average of 169 (**Supplementary Figure 1B**). While there is more variability in working with RP for the detection of genes, both platforms are consistent in finding an insufficient number of genes in mesPVAT. This prevented us from being able to draw meaningful and reliable conclusions from analyses and further suggests inherent properties of the rat mesPVAT, resulting in generally low RNA content. Nevertheless, this revised protocol collectively increased the nuclei yield across all fat depots and improved the detection of transcripts and genes in cow WAT.

We publish this additional sequencing information to initiate further discussion on the characteristics and challenges associated with transcriptomic analyses of these adipose depots and add to the knowledge already obtained.

## Data Availability

Raw and processed snRNAseq data are available on the Gene Expression Omnibus (GEO; https://www.ncbi.nlm.nih.gov/geo/), Life Science Identifier (LSID) accession ID GSE320334.

## References

[B1] Arduini A. Fleming S. J. Xiao L. Hall A. W. Akkad A.-D. Chaffin M. D. . (2025). Transcriptional profile of the rat cardiovascular system at single-cell resolution. Cell Rep. 44, 115091. doi: 10.1016/j.celrep.2024.115091, PMID: 39709602 PMC11781962

[B2] Caesar R. Drevon C. A. (2008). Pancreatic contamination of mesenteric adipose tissue samples can be avoided by adjusted dissection procedures. J. Lipid Res. 49, 1588–1594. doi: 10.1194/jlr.d800013-jlr200, PMID: 18408254

[B3] Chi Z. Jia Q. Yang H. Ren H. Jin C. He J. . (2024). snRNA-seq of adipose tissues reveals the potential cellular and molecular mechanisms of cold and disease resistance in Mongolian cattle. BMC Genomics 25, 999. doi: 10.1186/s12864-024-10913-y, PMID: 39448899 PMC11520132

[B4] Chirivi M. Abou-Rjeileh U. Myers M. Parales-Giron J. Worden L. Lock A. L. . (2025). TLR4 and prostaglandin pathways at the crossroads of endotoxemia-induced lipolysis. Front. Immunol. 16. doi: 10.3389/fimmu.2025.1591210, PMID: 40458405 PMC12127734

[B5] Cirera S. (2013). Highly efficient method for isolation of total RNA from adipose tissue. BMC Res. Notes. 6, 472. doi: 10.1186/1756-0500-6-472, PMID: 24245791 PMC4225616

[B6] Emont M. P. Jacobs C. Essene A. L. Pant D. Tenen D. Colleluori G. . (2022). A single-cell atlas of human and mouse white adipose tissue. Nature 603, 926–933. doi: 10.1038/s41586-022-04518-2, PMID: 35296864 PMC9504827

[B7] Gayoso A. Lopez R. Xing G. Boyeau P. Valiollah Pour Amiri V. Hong J. . (2022). A Python library for probabilistic analysis of single-cell omics data. Nat. Biotechnol. 40, 163–166. doi: 10.1038/s41587-021-01206-w, PMID: 35132262

[B8] Germain P.-L. Lun A. Garcia Meixide C. Macnair W. Robinson M. D. (2021). Doublet identification in single-cell sequencing data using scDblFinder. F1000Research 10, 979. doi: 10.12688/f1000research.73600.2, PMID: 35814628 PMC9204188

[B9] Han B. Li H. Zheng W. Zhang Q. Chen A. Zhu S. . (2025). A multi-tissue single-cell expression atlas in cattle. Nat. Genet. 57, 2546–2561. doi: 10.1038/s41588-025-02329-5, PMID: 40913183 PMC12513844

[B10] Hemmrich K. Denecke B. Paul N. E. Hoffmeister D. Pallua N. (2010). RNA isolation from adipose tissue: an optimized procedure for high RNA yield and integrity. Lab. Med. 41, 104–106. doi: 10.1309/LMFSBPUOA19MH5BV, PMID: 41345977

[B11] Heumos L. Schaar A. C. Lance C. Litinetskaya A. Drost F. Zappia L. . (2023). Best practices for single-cell analysis across modalities. Nat. Rev. Genet. 24, 550–572. doi: 10.1038/s41576-023-00586-w, PMID: 37002403 PMC10066026

[B12] Kersey H. N. Acri D. J. Dabin L. C. Hartigan K. Mustaklem R. Park J. H. . (2025). Comparative analysis of nuclei isolation methods for brain single-nucleus RNA sequencing. bioRxiv, 2025.2003.2025.645306. doi: 10.1101/2025.03.25.645306, PMID: 41875869 PMC13030979

[B13] Kim N. Kang H. Jo A. Yoo S.-A. Lee H.-O. (2023). Perspectives on single-nucleus RNA sequencing in different cell types and tissues. J. Pathol. Trans. Med. 57, 52–59. doi: 10.4132/jptm.2022.12.19, PMID: 36623812 PMC9846005

[B14] Kotzbeck P. Giordano A. Mondini E. Murano I. Severi I. Venema W. . (2018). Brown adipose tissue whitening leads to brown adipocyte death and adipose tissue inflammation. J. Lipid Res. 59, 784–794. doi: 10.1194/jlr.M079665, PMID: 29599420 PMC5928436

[B15] Kurabo Industries Ltd . (2006). Technical datasheet: *24RNA Tissue Total RNA Kit (Product Code 24RNA-Tissue-604).* (Japan). Available online at: https://www.kurabo.co.jp/bio/English/pdf/24RNA-Tissue-604.pdf (Accessed March 18, 2026).

[B16] Lake B. B. Chen S. Sos B. C. Fan J. Kaeser G. E. Yung Y. C. . (2018). Integrative single-cell analysis of transcriptional and epigenetic states in the human adult brain. Nat. Biotechnol. 36, 70–80. doi: 10.1038/nbt.4038, PMID: 29227469 PMC5951394

[B17] Michelotti T. C. Kisby B. R. Flores L. S. Tegeler A. P. Fokar M. Crasto C. . (2022). Single-nuclei analysis reveals depot-specific transcriptional heterogeneity and depot-specific cell types in adipose tissue of dairy cows. Front. Cell Dev. Biol. 10, 1025240. doi: 10.3389/fcell.2022.1025240, PMID: 36313560 PMC9616121

[B18] National Academies of Sciences, Engineering, and Medicine Division on Earth and Life Studies Board on Agriculture and Natural Resources Committee on Nutrient Requirements of Dairy Cattle (2021). Nutrient requirements of dairy cattle: eighth revised edition. (Washington (DC): National Academies Press (US). Available online at: http://www.ncbi.nlm.nih.gov/books/NBK600603/ (Accessed March 18, 2026). 38386771

[B19] National Research Council (US), Committee for the Update of the Guide for the Careand Use of Laboratory Animals . (2011). Guide for the Care and Use of Laboratory Animals[Internet], eighth ed., (The National Academies Collection: Reports funded byNational Institutes of Health). (Washington, DC, US: National Academies Press). Available online at: Available at: http://www.ncbi.nlm.nih.gov/books/NBK54050/ (Accessed March 18, 2026).

[B20] Rosell M. Kaforou M. Frontini A. Okolo A. Chan Y.-W. Nikolopoulou E. . (2014). Brown and white adipose tissues: Intrinsic differences in gene expression and response to cold exposure in mice. Am. J. Physiol. - Endocrinol. Metab. 306, E945–E964. doi: 10.1152/ajpendo.00473.2013, PMID: 24549398 PMC3989735

[B21] Slyper M. Porter C. B. M. Ashenberg O. Waldman J. Drokhlyansky E. Wakiro I. . (2020). A single-cell and single-nucleus RNA-Seq toolbox for fresh and frozen human tumors. Nat. Med. 26, 792–802. doi: 10.1038/s41591-020-0844-1, PMID: 32405060 PMC7220853

[B22] So J. Strobel O. Wann J. Kim K. Paul A. Acri D. J. . (2025). Robust single-nucleus RNA sequencing reveals depot-specific cell population dynamics in adipose tissue remodeling during obesity. eLife 13, RP97981. doi: 10.7554/eLife.97981.3.sa4, PMID: 39804687 PMC11729396

[B23] Thompson J. M. Watts S. W. Terrian L. Contreras G. A. Rockwell C. Rendon C. J. . (2024). A cell atlas of thoracic aortic perivascular adipose tissue: A focus on mechanotransducers. Am. J. Physiol. Heart Circulatory Physiol. 326, H1252–H1265. doi: 10.1152/ajpheart.00040.2024, PMID: 38517229 PMC11380965

[B24] Truong D. Lamhamedi-Cherradi S. E. Ludwig J. A. (2020). Nuclei isolation for single-nuclei RNA sequencing. protocols.io. doi: 10.17504/protocols.io.bkacksaw, PMID: 41632027

[B25] Van Hauwaert E. L. Gammelmark E. Sárvári A. K. Larsen L. Nielsen R. Madsen J. G. S. . (2021). Isolation of nuclei from mouse white adipose tissues for single-nucleus genomics. STAR Protoc. 2, 100612. doi: 10.1016/j.xpro.2021.100612, PMID: 34189477 PMC8220393

[B26] Whytock K. L. Divoux A. Sun Y. Hopf M. Yeo R. X. Pino M. F. . (2023). Isolation of nuclei from frozen human subcutaneous adipose tissue for full-length single-nuclei transcriptional profiling. STAR Protoc. 4, 102054. doi: 10.1016/j.xpro.2023.102054, PMID: 36853719 PMC9876942

[B27] Wolf F. A. Angerer P. Theis F. J. (2018). SCANPY: Large-scale single-cell gene expression data analysis. Genome Biol. 19, 15. doi: 10.1186/s13059-017-1382-0, PMID: 29409532 PMC5802054

