# Supplementary material for: Nuclei isolation from rat and cow white adipose tissues for single-nucleus RNA sequencing; rat WAT remains a challenge
Source: Front Physiol. 2026 Mar 26;17:1741037. doi: 10.3389/fphys.2026.1741037 (PMC13063377; doi:10.3389/fphys.2026.1741037)
Supplement: Supplementary file 1 [file DataSheet1.docx]

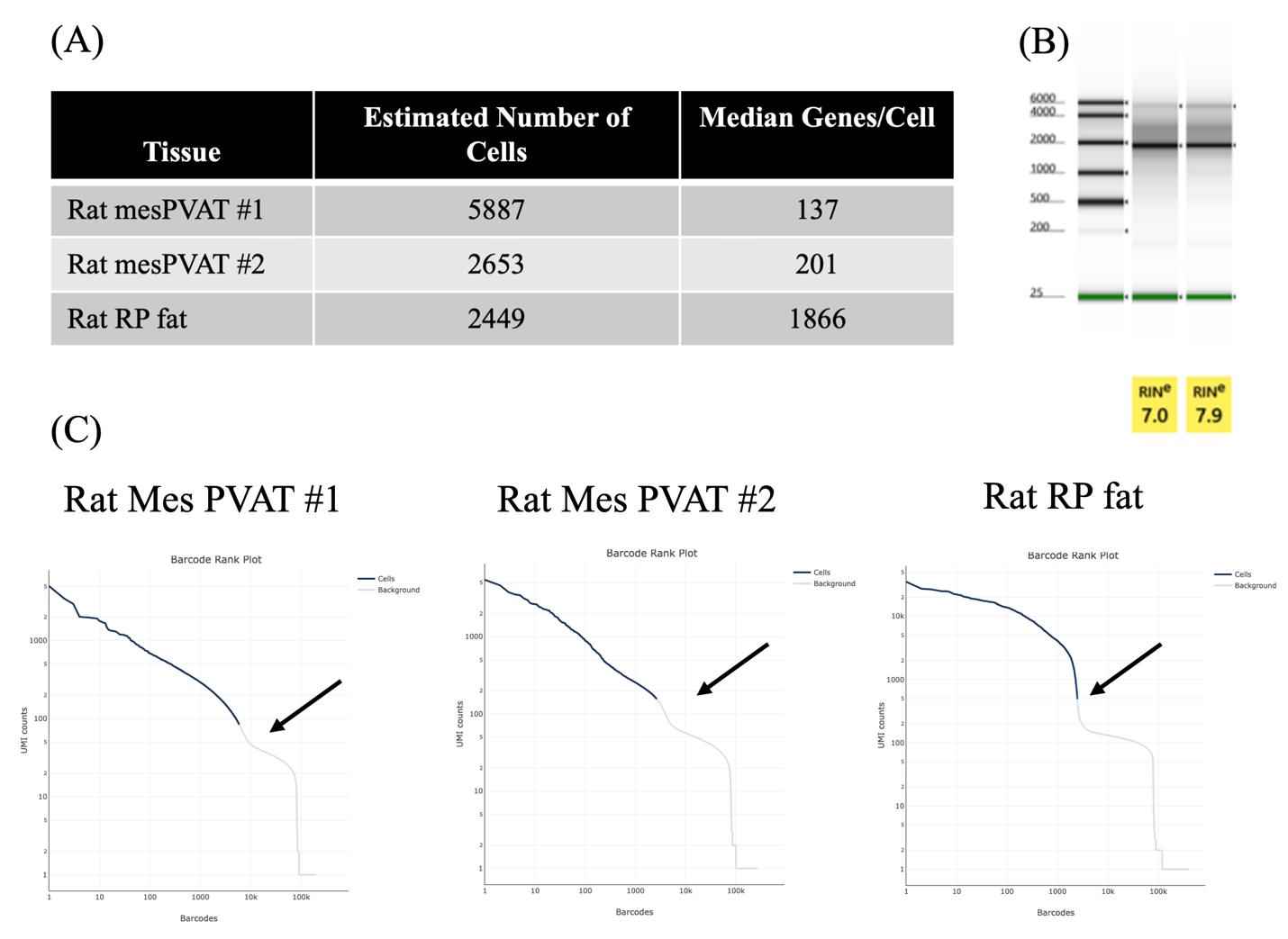


Supplemental Figure 1. (A) Estimated number of cells and median genes/cell for mesPVAT samples in (C). (B) RNA Integrity Number (RIN) values of RNA isolation from representative mesPVAT tissue samples. (C) Knee plots of mesPVAT snRNAseq data using the 10X Genomics Chromium Single Cells 3’ Reagent Kit v3.1 as outlined in supplemental methods. Arrows identify inflection point between real cells and background GEMs.


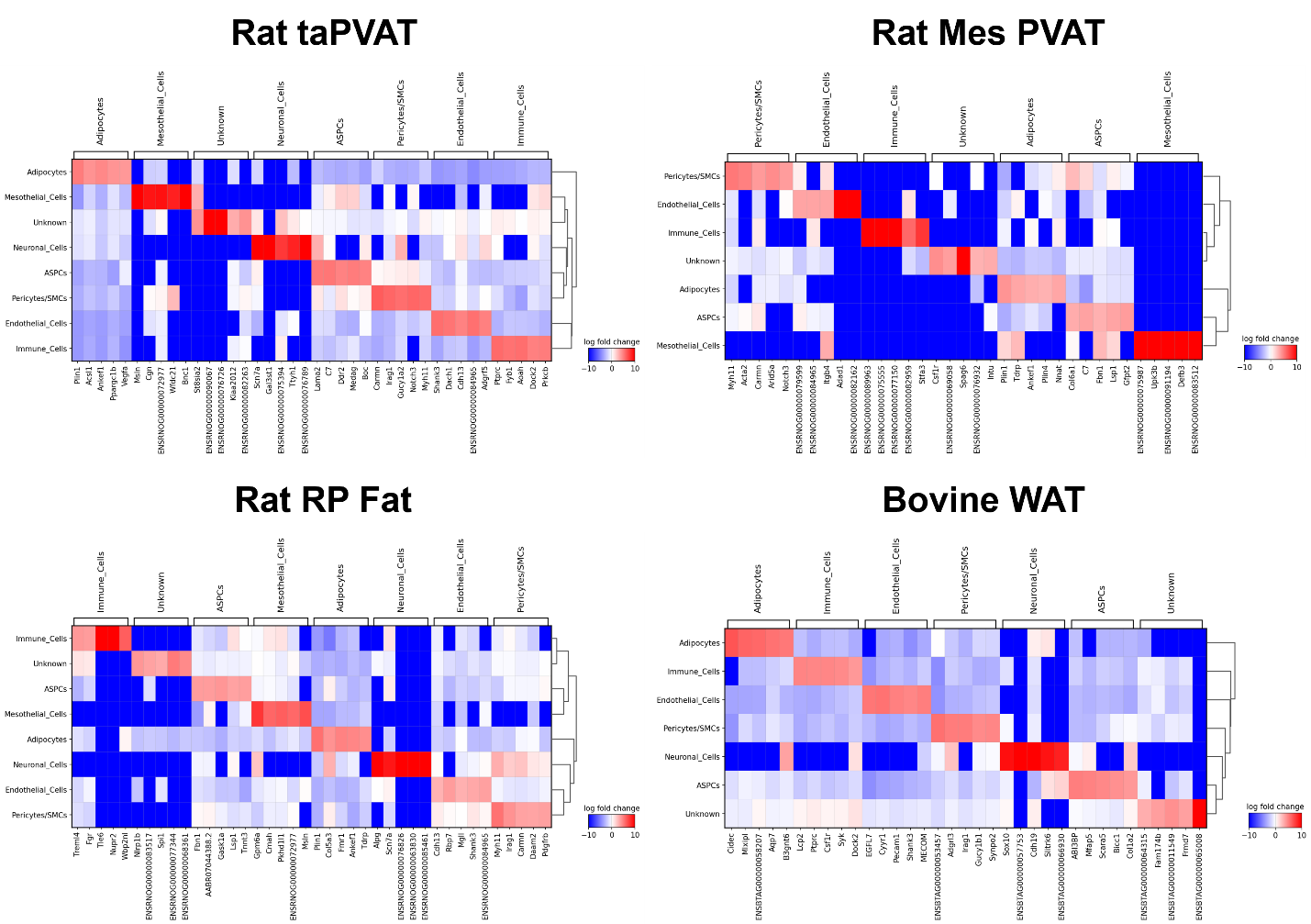
Supplemental Figure 2. Heatmap of the top 5 cell type cluster markers in each adipose tissue depots.
