# Supplementary material for: Nuclei isolation from rat and cow white adipose tissues for single-nucleus RNA sequencing; rat WAT remains a challenge
Source: Front Physiol. 2026 Mar 26;17:1741037. doi: 10.3389/fphys.2026.1741037 (PMC13063377; doi:10.3389/fphys.2026.1741037)
Supplement: Supplementary file 2 [file DataSheet2.docx]

**S1. Supplemental Methods**

**S1.1**  **Total RNA isolation and 10X Genomics single-nuclei sequencing**

Bovine and rat white adipose tissues were collected and processed for storage at -80ºC as described in section 2.1 “Tissue Collection” of the Methods section. For evaluation of RNA integrity following collection and storage, total RNA was isolated using the Qiagen RNeasy Plus Universal Kit and evaluated with the high sensitivity RNA assay on an Agilent 4200 TapeStation to obtain the RNA integrity number (RIN).

Nuclei from ~50 mg rat and bovine white adipose tissue were isolated following a previously published protocol for fatty tissues (Truong et al., 2020). Nuclei concentrations were determined with a Nexcelom Cellometer Vision and Viastain AO/PI staining solution at a 1:1 dilution. Nuclei were stained with DAPI, filtered in a 40 µm filter, then sorted for DAPI positive nuclei immediately before loading into a 10X Genomics 3’ v3.1 Chromium Chip. Single-nuclei library preparation using the 10X Chromium Next GEM Single Cell 3’ Reagent Kit v 3.1 (Dual Index) was performed according to manufacturer’s instructions ([CG000315](https://www.ncbi.nlm.nih.gov/nuccore/CG000315), Rev D) targeting a cell recovery of 10,000. Sequencing libraries were quantified using the KAPA Library Quantification Kit according to manufacturer’s recommended protocol with a standard melt curve run to ensure absence of adapter dimers. Libraries were sequenced at a target depth of 50,000 reads/nuclei on a NovaSeq6000 by Novogene. FastQC was used for initial quality control of sequencing reads and the 10X Genomics CellRanger v7.1.0 pipeline was utilized to align reads to the rat reference genome Rnor_6.0 (assembly GCA_000001895.4) and generate knee plots.

**S1.2 References**

Truong, D., Lamhamedi-Cherradi, S. E., and Ludwig, J. A. (2020). Nuclei Isolation for Single-Nuclei RNA sequencing. protocols.io. doi: 10.17504/protocols.io.bkacksaw
